# Analysis the molecular similarity of least common amino acid sites in ACE2 receptor to predict the potential susceptible species for SARS-CoV-2

**DOI:** 10.1371/journal.pone.0293441

**Published:** 2024-05-02

**Authors:** YeZhi Hu, Arivizhivendhan Kannan Villalan, Xin Fan, Shuang Zhang, Fekede Regassa Joka, XiaoDong Wu, HaoNing Wang, XiaoLong Wang

**Affiliations:** 1 Key Laboratory for Wildlife Diseases and Bio-security Management of Heilongjiang Province, Harbin, Heilongjiang Province, China; 2 College of Wildlife and Protected Area, Northeast Forestry University, Harbin, Heilongjiang Province, China; 3 Ethiopian Wildlife Conservation Authority, Addis Ababa, Ethiopian; 4 China Animal Health and Epidemiology Center, Qingdao, Shandong Province, China; 5 School of Geography and Tourism, Harbin University, Harbin, Heilongjiang Province, China; Jordan University of Science and Technology, JORDAN

## Abstract

SARS-CoV-2 infections in animals have been reported globally. However, the understanding of the complete spectrum of animals susceptible to SARS-CoV-2 remains limited. The virus’s dynamic nature and its potential to infect a wide range of animals are crucial considerations for a One Health approach that integrates both human and animal health. This study introduces a bioinformatic approach to predict potential susceptibility to SARS-CoV-2 in both domestic and wild animals. By examining genomic sequencing, we establish phylogenetic relationships between the virus and its potential hosts. We focus on the interaction between the SARS-CoV-2 genome sequence and specific regions of the host species’ ACE2 receptor. We analyzed and compared ACE2 receptor sequences from 29 species known to be infected, selecting 10 least common amino acid sites (LCAS) from key binding domains based on similarity patterns. Our analysis included 49 species across primates, carnivores, rodents, and artiodactyls, revealing complete consistency in the LCAS and identifying them as potentially susceptible. We employed the LCAS similarity pattern to predict the likelihood of SARS-CoV-2 infection in unexamined species. This method serves as a valuable screening tool for assessing infection risks in domestic and wild animals, aiding in the prevention of disease outbreaks.

## 1. Introduction

Corona virus disease 2019 (COVID-19) is a highly contagious zoonoses caused by the *Severe Acute Respiratory Syndrome Corona virus 2* (SARS-CoV-2). Since the first detection in Wuhan in December 2019, COVID-19 has rapidly spread globally [1], but the origin of the coronavirus is still unknown. Bats and pangolins have been considered possible natural hosts for SARS-CoV-2, but there is no conclusive evidence [2, 3]. The range of SARS-CoV-2 hosts not only humans but also expanding other mammals such as pet cats and minks, which were infected in March and April of 2020 in Belgium and Spain, respectively [3, 4]. Subsequently, SARS-CoV-2 infection was detected in ferrets, dogs, golden hamsters, white-tailed deer, rhesus macaques, tigers, lions and so on, as reported by the World Organization for Animal Health (WOAH) [5, 6]. An increasing number of mammals are infected with the new coronavirus, indicating the risk of cross-species transmission of SARS-CoV-2. Cross-species transmission of SARS-CoV-2 may lead to the evolution of new hosts and further spread of the virus [7]. This poses a serious threat to global public health and biodiversity.

The SARS-CoV-2 viral genome specifically binds to receptors on the surface of host cells, which is a key link in viral infection [8]. So far, the virus has been infecting new species consisting of a specific homologous target receptor capable of binding the SARS-CoV-2 genome. The recognition of SARS-CoV-2 receptors is an important determinant of its transmission between species [9–11]. The specific receptor of the new coronavirus is angiotensin-converting enzyme 2 (ACE2), which is widely expressed in animals as a cell surface receptor. The abundance of ACE2 receptors in any organs of the body, including the brain, heart, kidney, nasopharynx, lymph nodes, small intestine, colon, stomach, thymus, skin, spleen, bone marrow, liver, blood vessels, and oral and nasal mucosa, renders them susceptible to infection by SARS-CoV-2 [12–14].

The researcher has extensively studied SARS-CoV-2 in order to determine its host range [15, 16]. However, animals at high risk of contracting SARS-CoV-2 cannot be accurately predicted by phylogenetic relationships based on comparisons of the entire ACE2 gene [15, 17]. In-Vivo experiments animal infection provide the best opportunity to understand the susceptibility of SARS-CoV-2 across mammals [18]. However, conducting In-Vivo studies on a wide array of animals, particularly wildlife, presents a considerable complexity demanding increased manpower and resources. Additionally, ethical concerns arise when performing experiments on the diverse range of wild animals. Therefore, our attention has been turned to the analysis of the key binding domain of ACE2 to SARS-CoV-2 to predict the high-risk susceptible animals [10, 19–24].The analysis of receptor similarity methods is often used to predict the transmission of the virus between species [25]. Myeongji Cho’s sequence-based approach suggests that it may be possible to identify virus transmission between hosts without requiring complex structural analysis [17]. This method has been used to study the host range of the new coronavirus by predicting the homology of receptor key amino acid sequences, and key binding site methods [15, 16, 26, 27]. On this basis, we proposed a new screening approach that involved screening and combining the important Last Common Amino acid Sites (LCAS) in ACE2 from known susceptible hosts, which served as a standard method to evaluate the risk of SARS-CoV-2 infection with unknown species. It can be used as a screening tool and has important scientific implications for discovering potential susceptible hosts of the SARS-CoV-2 virus and assessing its possible transmissibility across species.

## 2. Materials and methods

### 2.1 SARS-CoV-2 susceptible host collection

Reported SARS-CoV-2 infected species information were collected from the World Organization for Animal Health (WOAH) (https://www.woah.org/en/what-we-offer/emergency-preparedness/covid-19/) and literature [5, 28–31]. The naturally infected host species and experimentally infected host species information were separately summarized to understand the primary distribution of SARS-CoV-2 infection.

### 2.2 ACE2 receptor sequence collection

The protein sequences of ACE2 from mammalian species were gathered from the National Center for Biotechnology Information (NCBI) Protein Database (https://www.ncbi.nlm.nih.gov/) and Uniprot (UniProt). Queried for records containing “ACE2” as gene name and “Mammalia” as taxonomic class. Next, for selection by taxon, one complete ACE2 amino acid sequence per species was retained and extracted in FASTA format. Then, for sequence files, protein IDs were renamed as follow: ACE2_NCBI gene accession ID_ Species name.

### 2.3 ACE2 receptor data processing

The downloaded sequence file in FASTA format was imported into MAFFT [32] for sequence alignment and duplicate sequences were removed. Output in the same FASTA format. Then import the aligned sequences into BioEdit [33]. Find the human ACE2 receptor sequence in the sequence file and drag it to the first line. Using the human ACE2 sequence as a reference, delete sequences with missing or additional amino acid sites. Finally, rename the sequences, naming them with ’species_ sequence number’. All data were output in FASTA format.

### 2.4 LCAS selection

The collected ACE2 sequence species were distinguished into two parts: known susceptible species and unknown species. The key amino acid region of the human ACE2 receptor sequence that strongly binds to SARS-CoV-2 was screened from the literature [9, 10, 15, 19, 20, 34, 35]. Import the amino acid sequences of known susceptible species into BioEdit [33] and highlight the sites of the key amino acid domains that are screened out. Then paste the highlighted amino acid sites into a new Excel spreadsheet. Finally, using the human ACE2 receptor amino acid sequence as a standard, select the amino acid sites that are completely identical in all known species, which are the least common amino acid sites (LCAS). Documented the finalized LCAS set in an organized format for subsequent analyses. This comprehensive selection of amino acid sites represents the least common denominators across susceptible species, forming a robust foundation for further investigations.

### 2.5 Analysis of potentially susceptible hosts

The ACE2 sequences of unknown species was imported into BioEdit tool and highlighted the LCAS (Least Common Amino acid Sites) sites. The identical pattern of LCAS amino acid sites of known susceptibility were compared and analyzed with unknown species sequence into a new Excel spreadsheet for systematic analysis. Species displayed entirely identical LCAS patterns were categorized as potentially vulnerable hosts; nonidentical sequence species were categorized as non-potential susceptible hosts.

The MEGA11 software adjacency method (Neighbor Joining Method NJ) was used to construct a phylogenetic tree of potentially susceptible hosts. The average distance of each species in the NJ phylogenic tree was constructed between 0 and 1. We perform a bootstrap test with 1000 replicates to build a phylogenetic tree.

## 3. Result

### 3.1 Collection of SARS-CoV-2 susceptible hosts

**Fig 1:**
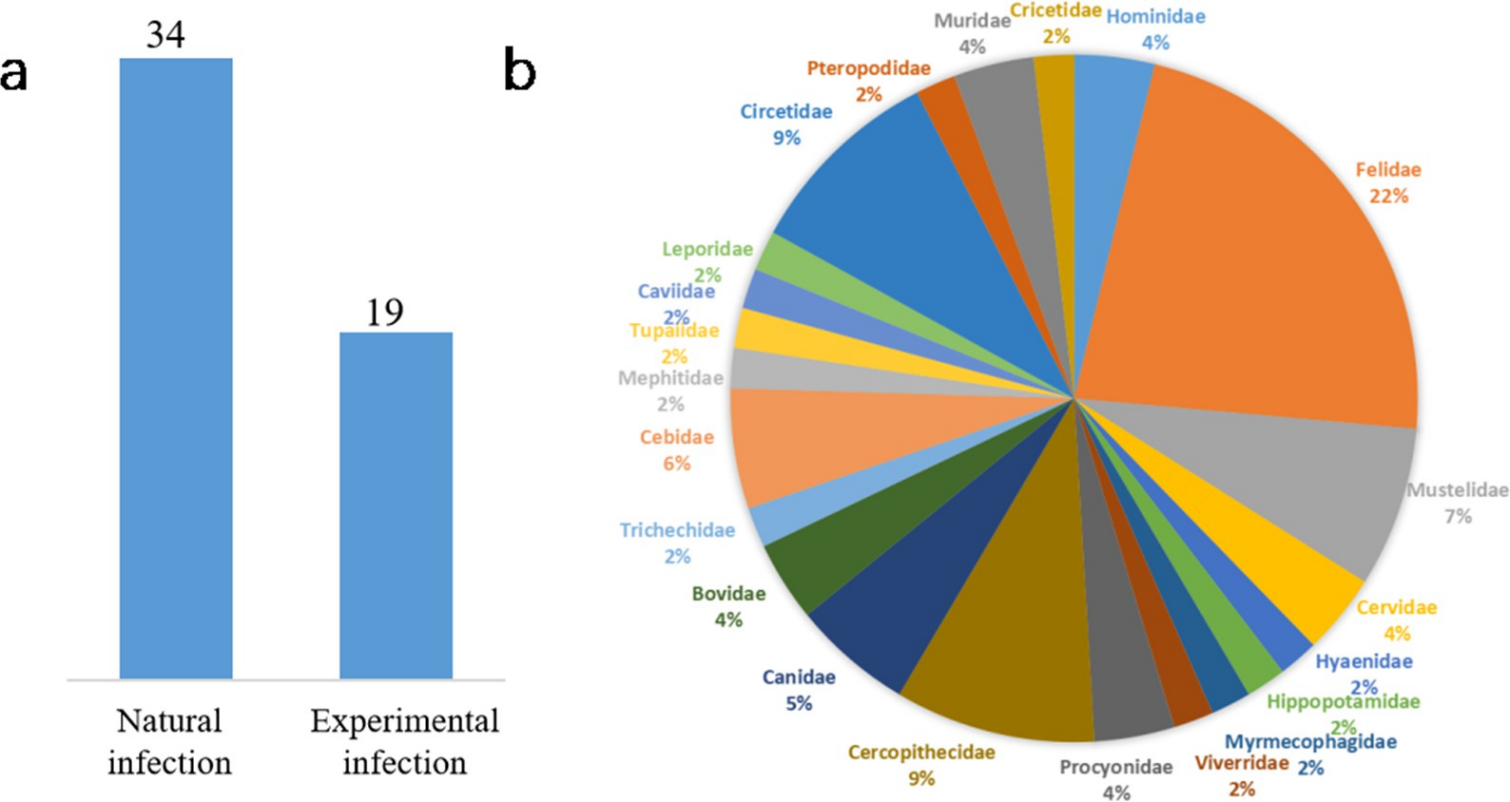
(a) COVID-19 reported species infected by natural and experimental condition and (b) Percentage of animal species in families infected with COVID-19.

The list of animals infected with SARS-CoV-2 was collected from WOAH reports and literature. The results reveal that a total of 63 species were infected with SARS-CoV-2, including 38 species from 16 families that were infected from natural sources (Table 1) and 25 species from 12 families that were infected under experimental conditions (Table 2). Known susceptibility host statistics (Fig 1).

**Table 1:** Animals naturally infected with SARS‐CoV‐2.

Family	Genus	Species	Reference
*Hominidae*	*Homo*	*Homo sapiens*	[36]
	*Gorilla*	*Gorilla gorilla gorilla*	[36]
*Felidae*	*Felis*	*Felis catus*	[36]
	*Puma*	*Puma concolor*	[36]
	*Panthera*	*Panthera uncia*	[36]
	*Prionailurus*	*Prionailurus viverrinus*	[36]
	*Panthera*	*Panthera tigris jacksoni*	[37]
		*Panthera leo persica*	[37]
		*Panthera pardus*	[38]
		*Panthera tigris*	[37]
		*Panthera leo*	[36]
	*Acinonyx*	*Acinonyx jubatus*	[37]
	*Lynx*	*Lynx lynx*	[36]
		*Lynx canadensis*	[36]
*Mustelidae*	*Neovison*	*Neovison vison*	[36]
	*Mustela*	*Mustela putorius furo*	[39]
	*Aonyx*	*Aonyx cinerea*	[36]
	*Lutra*	*Lutra lutra*	[36]
*Cervidae*	*Odocoileus*	*Odocoileus virginianus*	[36]
		*Odocoileus hemionus*	[36]
*Hyaenidae*	*Crocuta*	*Crocuta crocuta*	[36]
*Hippopotamidae*	*Hippopotamus*	*Hippopotamus amphibius*	[36]
*Myrmecophagidae*	*Myrmecophaga*	*Myrmecophaga tridactyla*	[36]
*Viverridae*	*Arctictis*	*Arctictis binturong*	[36]
*Procyonidae*	*Nasuella*	*Nasuella olivacea*	[36]
	*Nasua*	*Nasua nasua*	[40]
*Cercopithecidae*	*Mandrillus*	*Mandrillus sphinx*	[36]
*Canidae*	*Canis*	*Canis lupus familiaris*	[36]
	*Vulpes*	*Vulpes vulpes*	[36]
*Bovidae*	*Bos*	*Bos taurus*	[41]
	*Capra*	*Capra hircus*	[42]
*Trichechidae*	*Trichechus*	*Trichechus manatus manatus*	[36]
*Atelidae*	*Ateles*	*Ateles fuscieps*	[40]
	*Lagothrix*	*Lagothrix lagothricha*	[40]
*Rhinocerotidae*	*Ceratitherium*	*Ceratitherium simum*	[40]
*Cebidae*	*Saimiri*	*Saimiri sciureus*	[36]
	*Mico*	*Mico leucippe*	[36]
		*Mico melanurus*	[30]

**Table 2:** Animals experimentally infected with SARS‐CoV‐2.

Family	Genus	Species	Reference
*Mephitidae*	*Mephitis*	*Mephitis mephitis*	[31]
*Procyonidae*	*Procyon*	*Procyon lotor*	[31]
*Tupaiidae*	*Tupaia*	*Tupaia belangeri chinesis*	[43]
*Canidae*	*Nyctereutes*	*Nyctereutes procyonoides*	[44]
	*Canis*	*Canis latrans*	[40]
*Caviidae*	*Cavia*	*Cavia porcellus*	[5]
*Leporidae*	*Oryctolagus*	*Oryctolagus cuniculus*	[45]
*Circetidae*	*Mesocricetus*	*Mesocricetus auratus*	[46]
	*Cricetulus*	*Cricetulus griseus*	[47]
	*Phodopus*	*Phodopus sungorus*	[28]
		*Phodopus campbelli*	[28]
		*Phodopus roborovskii*	[28]
	*Myodes*	*Myodes glareolus*	[40]
	*Neotoma*	*Neotoma cinerea*	[28]
*Cercopithecidae*	*Chlorocebus*	*Chlorocebus aethiops*	[48]
	*Macaca*	*Macaca fascicularis*	[36]
		*Macaca mulatta*	[49]
	*Papio*	*Papio hamadryas*	[28]
	*Culicoides*	*Culicoides sonorensis*	[40]
*Pteropodidae*	*Rousettus*	*Rousettus leschenaultii*	[39]
		*Rousettus aegyptiacus*	[40]
*Muridae*	*Peromyscus*	*Peromyscus leucopus*	[50]
		*Peromyscus maniculatus*	[31]
*Danionidae*	*Danio*	*Danio rerio*	[40]
*Cebidae*	*Callithrix*	*Callithrix jacchus*	[40]

### 3.2 Collection of the ACE2 receptor sequence

We collected 407 ACE2 protein receptor sequences from various species from the Uniprot database. We scrutinized 86 complete ACE2 protein sequences after eliminating incomplete and duplicate sequences. In addition, we obtained 23 complete ACE2 protein sequences from the NCBI database. Finally, 109 ACE2 protein sequences from 45 families were selected for further evaluation to predict the potential risk host for SARS-CoV-2 infection (Table 3).

**Table 3:** List of ACE2 receptor sequences species used for prediction.

Family	Genus	Species	Sequences
*Hominidae*	*Homo*	*Homo sapiens*	Q9BYF1
	*Pongo*	*Pongo abelii*	H2PUZ5
	*Gorilla*	*Gorilla gorilla*	G3QWX4
	*Pan*	*Pan paniscus*	A0A2R9BKD8
		*Pan troglodytes*	A0A2J8KU96
*Cercopithecidae*	*Papio*	*Papio anubis*	A0A096N4X9
	*Cercocebus*	*Cercocebus atys*	A0A2K5KSD8
	*Macaca*	*Macaca mulatta*	F7AH40
		*Macaca fascicularis*	A0A2K5X283
		*Macaca nemestrina*	A0A2K6D1N8
	*Mandrillus*	*Mandrillus leucophaeus*	A0A2K5ZV99
	*Theropithecus*	*Theropithecus gelada*	XP_025227847
	*Piliocolobus*	*Piliocolobus tephrosceles*	A0A8C9GER2
	*Rhinopithecus*	*Rhinopithecus roxellana*	A0A2K6NFG7
	*Chlorocebus*	*Chlorocebus sabaeus*	A0A0D9RQZ0
		*Chlorocebus aethiops*	AAY57872
	*Colobus*	*Colobus angolensis*	A0A2K5JE65
*Felidae*	*Felis*	*Felis catus*	Q56H28
	*Neofelis*	*Neofelis diardi*	A0A7G6KLV6
	*Lynx*	*Lynx canadensis*	A0A667IF49
		*Lynx pardinus*	A0A485NF12
	*Panthera*	*Panthera pardus*	A0A6P4TH77
		*Panthera leo*	A0A8C8Y6V3
		*Panthera uncia*	XP_049499444
	*Acinonyx*	*Acinonyx jubatus*	A0A6J1YZV2
	*Puma*	*Puma concolor*	A0A6P6IQM4
		*Puma yagouaroundi*	XP_040324138
	*Prionailurus*	*Prionailurus viverrinus*	XP_047700804
		*Prionailurus bengalensis*	XP_043425608
*Mustelidae*	*Neovison*	*Neovison vison*	A0A7T0Q2W2
	*Mustela*	*Mustela pulourius*	Q2WG88
		*Mustela nigripes*	A0A7G6KLV4
		*Mustela erminea*	XP_032187677
	*Melogale*	*Melogale moschata*	A0A7D5FYI0
	*Arctonyx*	*Arctonyx collaris*	A0A7D5FU09
	*Enhydra*	*Enhydra lutris*	A0A2Y9KLV0
*Canidae*	*Canis*	*Canis lupus dingo*	A0A8C0JTU4
	*Nyctereutes*	*Nyctereutes procyonoides*	B4XEP4
	*Vulpes*	*Vulpes vulpes*	A0A3Q7RAT9
	*Chrysocyon*	*Chrysocyon brachyurus*	A0A7G6KLV7
	*Speothos*	*Speothos venaticus*	A0A7G6KLV5
*Circetidae*	*Peromyscus*	*Peromyscus maniculatus*	A0A6I9KY05
	*Phodopus*	*Phodopus sungorus*	A0A7T0LP11
		*Phodopus roborovskii*	A0A7T0PYW5
	*Mesocricetus*	*Mesocricetus auratus*	A0A1U7QTA1
	*Cricetulus*	*Cricetulus griseus*	XP_003503283
	*Microtus*	*Microtus ochrogaster*	A0A8J6FZ33
		*Microtus oregoni*	XP_041495910
	*Arvicola*	*Arvicola amphibius*	XP_038172229
*Cebidae*	*Cebus*	*Cebus imitator*	A0A2K5PYM0
	*Saimiri*	*Saimiri boliviensis*	A0A2K6SBD4
	*Sapajus*	*Sapajus apella*	A0A6J3II99
	*Callithrix*	*Callithrix jacchus*	F7CNJ6
*Camelidae*	*Lama*	*Lama glama*	A0A8F0WA13
	*Camelus*	*Camelus dromedarius*	A0A5N4C2M1
		*Camelus ferus*	XP_006194263
		*Camelus bactrianus*	XP_010966303
*Equidae*	*Equus*	*Equus caballus*	F6V9L3
		*Equus przewalskii*	XP_008542995
		*Equus asinus*	A0A8C4KQS2
		*Equus quagga*	XP_046528602
*Hylobatidae*	*Nomascus*	*Nomascus leucogenys*	G1RE79
	*Hylobates*	*Hylobates moloch*	XP_032612508
*Hyaenidae*	*Crocuta*	*Crocuta crocuta*	A0A6G1ARU3
*Otariidae*	*Callorhinus*	*Callorhinus ursinus*	A0A3Q7N3M7
	*Eumetopias*	*Eumetopias jubatus*	XP_027970822
	*Zalophus*	*Zalophus californianus*	A0A6J2EID0
*Manidae*	*Manis*	*Manis pentadactyla*	A0A7D5TP47
		*Manis javanica*	XP_017505746
*Pteropodidae*	*Rousettus*	*Rousettus leschenaultia*	D8WU01
		*Rousettus aegyptiacus*	A0A7J8EHI0
*Rhinolophidae*	*Rhinolophus*	*Rhinolophus macrotis*	E2DHI3
		*Rhinolophus ferrumequinum*	A0A671F9Q9
*Ursidae*	*Ailuropoda*	*Ailuropoda melanoleuca*	A0A7N5K7A3
*Bovidae*	*Bos*	*Bos taurus*	Q58DD0
	*Capra*	*Capra hircus*	A0A452EVJ5
*Monodontidae*	*Monodon*	*Monodon monoceros*	A0A8C6FDA8
	*Delphinapterus*	*Delphinapterus leucas*	A0A2Y9M9H3
*Tarsiidae*	*Carlito*	*Carlito syrichta*	A0A1U7TY97
	*Condylura*	*Condylura cristata*	XP_012585871
*Cervidae*	*Odocoileus*	*Odocoileus virginianus*	A0A6J0Z472
*Chinchillidae*	*Chinchilla*	*Chinchilla lanigera*	A0A8C2UPB0
*Dipodidae*	*Jaculus*	*Jaculus jaculus*	A0A8C5JWR5
*Bathyergidae*	*Heterocephalus*	*Heterocephalus glaber*	A0A0N8EUX7
	*Fukomys*	*Fukomys damarensis*	XP_010643477
*Vombatidae*	*Vombatus*	*Vombatus ursinus*	A0A4X2M679
*Tayassuidae*	*Catagonus*	*Catagonus wagneri*	A0A8C3WSW9
*Orycteropodidae*	*Orycteropus*	*Orycteropus afer*	A0A8B7ASS9
*Viverridae*	*Paguma*	*Paguma larvata*	Q56NL1
*Elephantidae*	*Loxodonta*	*Loxodonta africana*	G3T6Q2
*Sciuridae*	*Sciurus*	*Sciurus vulgaris*	A0A8D2JNG0
*Balaenopteridae*	*Balaenoptera*	*Balaenoptera musculus*	A0A8B8WGR5
		*Balaenoptera acutorostrata*	A0A452CBT6
*Phocaenidae*	*Phocoena*	*Phocoena sinus*	A0A8C9CHJ8
*Physeteridae*	*Physeter*	*Physeter catodon*	XP_023971279
*Indriidae*	*Propithecus*	*Propithecus coquereli*	A0A2K6GHW5
*Heteromyidae*	*Dipodomys*	*Dipodomys ordii*	A0A1S3GHT7
*Leporidae*	*Oryctolagus*	*Oryctolagus cuniculus*	G1TEF4
*Muridae*	*Rattus*	*Rattus norvegicus*	Q5EGZ1
	*Grammomys*	*Grammomys surdaster*	XP_028617961
*Lipotidae*	*Lipotes*	*Lipotes vexillifer*	A0A340Y3Y6
*Phocidae*	*Neomonachus*	*Neomonachus schauinslandi*	A0A2Y9GEI9
*Aotidae*	*Aotus*	*Aotus nancymaae*	A0A2K5DQ16
*Spalacidae*	*Nannospalax*	*Nannospalax galili*	XP_008839098
*Tenrecidae*	*Echinops*	*Echinops telfairi*	XP_004710002
*Herpestidae*	*Suricata*	*Suricata suricatta*	A0A673UPR4
*Rhinocerotidae*	*Ceratotherium*	*Ceratotherium simum*	XP_004435206
*Lemuridae*	*Prolemur*	*Prolemur simus*	A0A8C8YW84
*Delphinidae*	*Tursiops*	*Tursiops truncatus*	A0A2U4AJL3

### 3.3 Processing of ACE2 receptor data

We classified 109 ACE2 receptor sequences by dividing them into two groups: the known vulnerable hosts group (29 species in 10 families) and the unknown susceptible hosts group (80 species in 35 families) (Tables 1 and 2). We screened 29 species of ACE2 receptor sequences from 109 as known to be sensitive to SARS-CoV-2. The key regions of the ACE2 receptor sequence in the human ACE2 receptor have been selected for further study (Table 4).

**Table 4:** Analysis the similarity of LCAS in conserved loci of known susceptible hosts.

Family	Species	19	20	24	27	28	30	31	34	35	37	38	41	42	45	53	68	79	82	83	90	322	325	330	353	354	355	357	393
*Hominidae*	*Homo sapiens*	**S**	T	Q	T	**F**	D	**K**	H	**E**	E	D	**Y**	Q	**L**	**N**	**K**	L	M	Y	N	N	Q	N	K	G	**D**	**R**	R
	*Gorilla gorilla gorilla*	**S**	T	Q	T	**F**	D	**K**	H	**E**	E	D	**Y**	Q	**L**	**N**	**K**	L	M	Y	N	N	Q	N	K	G	**D**	**R**	R
*Felidae*	*Panthera pardus*	**S**	T	L	T	**F**	E	**K**	H	**E**	E	E	**Y**	Q	**L**	**N**	**K**	L	T	Y	N	N	Q	N	K	G	**D**	**R**	R
	*Panthera leo*	**S**	T	L	T	**F**	E	**K**	H	**E**	E	E	**Y**	Q	**L**	**N**	**K**	L	T	Y	N	N	Q	N	K	G	**D**	**R**	R
	*Panthera uncia*	**S**	T	L	T	**F**	E	**K**	H	**E**	E	E	**Y**	Q	**L**	**N**	**K**	L	T	Y	N	N	Q	N	K	G	**D**	**R**	R
	*Felis catus*	**S**	T	L	T	**F**	E	**K**	H	**E**	E	E	**Y**	Q	**L**	**N**	**K**	L	T	Y	N	N	Q	N	K	G	**D**	**R**	R
	*Puma concolor*	**S**	T	L	T	**F**	E	**K**	H	**E**	E	E	**Y**	Q	**L**	**N**	**K**	L	T	Y	N	N	Q	N	K	G	**D**	**R**	R
	*Prionailurus viverrinus*	**S**	T	L	T	**F**	E	**K**	H	**E**	E	E	**Y**	Q	**L**	**N**	**K**	L	T	Y	N	N	Q	N	K	G	**D**	**R**	R
	*Lynx canadensis*	**S**	T	L	T	**F**	E	**K**	H	**E**	E	E	**Y**	Q	**L**	**N**	**K**	L	T	Y	N	N	Q	N	K	G	**D**	**R**	R
	*Acinonyx jubatus*	**S**	T	L	T	**F**	E	**K**	H	**E**	E	E	**Y**	Q	**L**	**N**	**K**	L	T	Y	N	N	Q	K	K	G	**D**	**R**	R
*Cercopithecidae*	*Chlorocebus sabaeus*	**S**	T	Q	T	**F**	D	**K**	H	**E**	E	D	**Y**	Q	**L**	**N**	**K**	L	M	Y	N	N	Q	N	K	G	**D**	**R**	R
	*Chlorocebus aethiops*	**S**	T	Q	T	**F**	D	**K**	H	**E**	E	D	**Y**	Q	**L**	**N**	**K**	L	M	Y	N	N	Q	N	K	G	**D**	**R**	R
	*Macaca fascicularis*	**S**	T	Q	T	**F**	D	**K**	H	**E**	E	D	**Y**	Q	**L**	**N**	**K**	L	M	Y	N	N	Q	N	K	G	**D**	**R**	R
	*Macaca mulatta*	**S**	T	Q	T	**F**	D	**K**	H	**E**	E	D	**Y**	Q	**L**	**N**	**K**	L	M	Y	N	N	Q	N	K	G	**D**	**R**	R
*Canidae*	*Vulpes vulpes*	**S**	-	L	T	**F**	E	**K**	Y	**E**	E	E	**Y**	Q	**L**	**N**	**K**	L	T	Y	D	N	Q	N	K	G	**D**	**R**	R
	*Nyctereutes procyonoides*	**S**	-	L	T	**F**	E	**K**	Y	**E**	E	E	**Y**	Q	**L**	**N**	**K**	L	T	Y	D	N	Q	N	R	G	**D**	**R**	R
*Hyaenidae*	*Crocuta crocuta*	**S**	T	L	T	**F**	E	**K**	Y	**E**	Q	E	**Y**	L	**L**	**N**	**K**	L	T	Y	D	N	Q	N	K	G	**D**	**R**	K
*Mustelidae*	*Neovison vison*	**S**	T	L	T	**F**	E	**K**	Y	**E**	E	E	**Y**	Q	**L**	**N**	**K**	H	T	Y	D	N	E	N	K	H	**D**	**R**	R
	*Mustela putorius furo*	**S**	T	L	T	**F**	E	**K**	Y	**E**	E	E	**Y**	Q	**L**	**N**	**K**	H	T	Y	D	N	E	N	K	R	**D**	**R**	R
	*Mustela erminea*	**S**	T	L	T	**F**	E	**K**	Y	**E**	E	E	**Y**	Q	**L**	**N**	**K**	H	T	Y	D	N	E	N	K	R	**D**	**R**	R
*Cervidae*	*Odocoileus virginianus*	**S**	T	Q	T	**F**	E	**K**	H	**E**	E	D	**Y**	Q	**L**	**N**	**K**	M	T	Y	N	H	Q	N	K	G	**D**	**R**	R
*Circetidae*	*Cricetulus griseus*	**S**	I	Q	T	**F**	D	**K**	Q	**E**	E	D	**Y**	Q	**L**	**N**	**K**	L	N	Y	N	H	Q	N	K	G	**D**	**R**	R
	*Phodopus roborovskii*	**S**	I	Q	S	**F**	D	**K**	Q	**E**	E	D	**Y**	Q	**L**	**N**	**K**	L	N	Y	N	H	K	N	K	E	**D**	**R**	R
	*Mesocricetus auratus*	**S**	I	Q	T	**F**	D	**K**	Q	**E**	E	D	**Y**	Q	**L**	**N**	**K**	L	N	Y	N	Y	Q	N	K	G	**D**	**R**	R
	*Phodopus sungorus*	**S**	I	Q	T	**F**	D	**K**	Q	**E**	E	D	**Y**	Q	**L**	**N**	**K**	L	N	Y	N	H	K	N	K	E	**D**	**R**	R
	*Peromyscus maniculatus*	**S**	I	Q	I	**F**	D	**K**	Q	**E**	E	D	**Y**	Q	**L**	**N**	**K**	L	N	Y	N	H	Q	N	K	G	**D**	**R**	R
*Bovidae*	*Bos taurus*	**S**	T	Q	T	**F**	E	**K**	H	**E**	E	D	**Y**	Q	**L**	**N**	**K**	M	T	Y	N	Y	Q	N	K	G	**D**	**R**	R
	*Capra hircus*	**S**	T	Q	T	**F**	E	**K**	H	**E**	E	D	**Y**	Q	**L**	**N**	**K**	M	T	Y	N	Y	Q	N	K	G	**D**	**R**	R
*Leporidae*	*Oryctolagus cuniculus*	**S**	T	L	T	**F**	E	**K**	Q	**E**	E	D	**Y**	Q	**L**	**N**	**K**	L	T	Y	N	S	Q	N	K	G	**D**	**R**	R

### 3.4 Screening of LCAP

**Fig 2:**
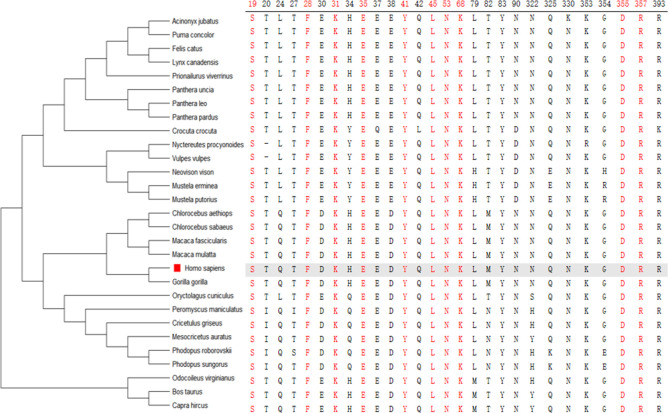
The key structural domains in ACE2 from known SARS-CoV-2 susceptible species.

The key regions of the ACE2 receptor sequence in the human ACE2 receptor was compared to the known susceptible to SARS-CoV-2 (Table 4). As a result of the comparison, the 10 most common amino acid sites—19, 28, 31, 35, 41, 45, 53, 68, 355 and 357—were identified and used them to further screen the potential risk host for SARS-CoV-2 (Fig 2).

### 3.5 Analysis of potentially susceptible hosts

**Fig 3:**
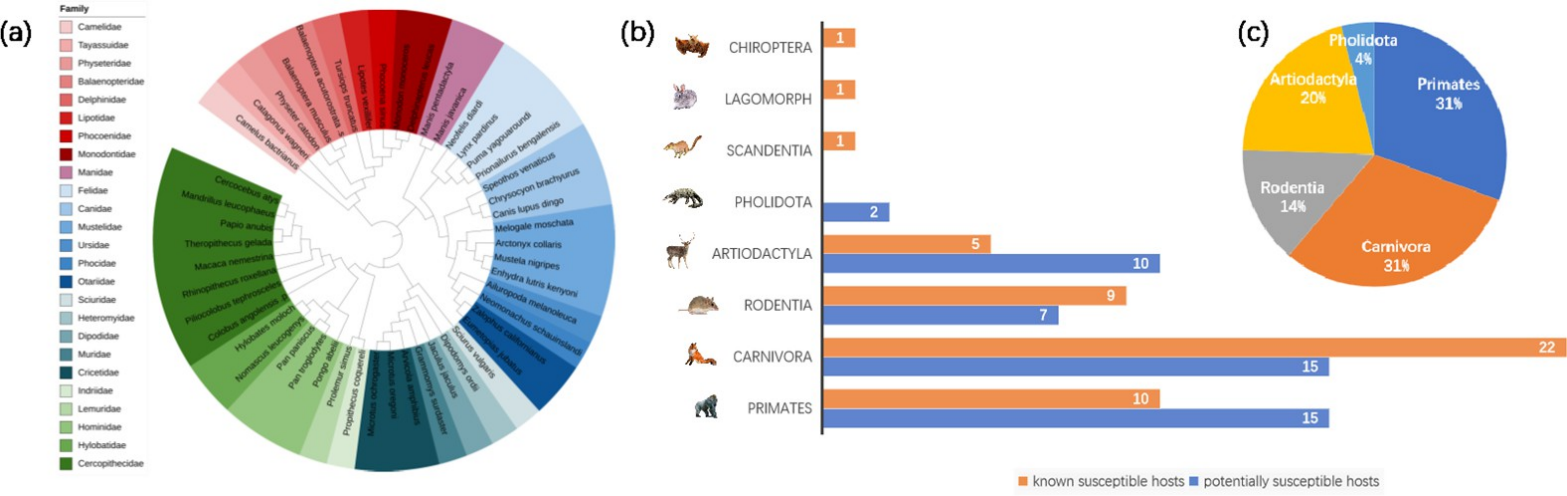
(a) The MEGA11 module calculates the IQ-TREE optimal model to build a phylogenetic tree. iTOL shows the percentage of the total number of species in the outer circle by order, including proportion, and the number of species in the inner circle by family. (b) Shows the number of species in each order in a two-dimensional bar chart. (c) Percentage of animal species in the classification orders potential risk for COVID 19.

In this study, ACE sequences from 80 unknown species were compared to 10 LCAS, and their similarity pattern was examined. The ACE2 receptor sequences of 49 species across25 families were entirely similar to the 10 LCAS of known sensitive species, suggesting their potential susceptibility to SARS-CoV-2 (Table 5). Thirty-one species from 21 families were considered non-potential susceptible hosts because they were not related to the 10 LCAS (Table 6). Potential susceptible hosts are primarily located in the orders Primates, Carnivora, Rodentia, and Artiodactyla, indicating that closely related animals are more likely to be infected with the novel coronavirus. It illustrates the evolutionary links between potentially susceptible risk hosts (Fig 3).

**Table 5:** LCAS of potentially susceptible hosts.

Order	Family	Species	19	28	31	35	41	45	53	68	355	357
*Primates*	*Hominidae*	*Pongo abelii*	S	F	K	E	Y	L	N	K	D	R
		*Pan troglodytes*	S	F	K	E	Y	L	N	K	D	R
		*Pan paniscus*	S	F	K	E	Y	L	N	K	D	R
	*Cercopithecidae*	*Papio anubis*	S	F	K	E	Y	L	N	K	D	R
		*Cercocebus atys*	S	F	K	E	Y	L	N	K	D	R
		*Macaca nemestrina*	S	F	K	E	Y	L	N	K	D	R
		*Rhinopithecus roxellana*	S	F	K	E	Y	L	N	K	D	R
		*Piliocolobus tephrosceles*	S	F	K	E	Y	L	N	K	D	R
		*Mandrillus leucophaeus*	S	F	K	E	Y	L	N	K	D	R
		*Theropithecus gelada*	S	F	K	E	Y	L	N	K	D	R
		*Colobus angolensis palliatus*	S	F	K	E	Y	L	N	K	D	R
	*Hylobatidae*	*Nomascus leucogenys*	S	F	K	E	Y	L	N	K	D	R
		*Hylobates moloch*	S	F	K	E	Y	L	N	K	D	R
	*Lemuridae*	*Prolemur simus*	S	F	K	E	Y	L	N	K	D	R
	*Indriidae*	*Propithecus coquereli*	S	F	K	E	Y	L	N	K	D	R
*Carnivora*	*Felidae*	*Lynx pardinus*	S	F	K	E	Y	L	N	K	D	R
		*Puma yagouaroundi*	S	F	K	E	Y	L	N	K	D	R
		*Prionailurus bengalensis*	S	F	K	E	Y	L	N	K	D	R
		*Neofelis diardi*	S	F	K	E	Y	L	N	K	D	R
	*Ursidae*	*Ailuropoda melanoleuca*	S	F	K	E	Y	L	N	K	D	R
	*Canidae*	*Canis lupus dingo*	S	F	K	E	Y	L	N	K	D	R
		*Speothos venaticus*	S	F	K	E	Y	L	N	K	D	R
		*Chrysocyon brachyurus*	S	F	K	E	Y	L	N	K	D	R
	*Mustelidae*	*Mustela nigripes*	S	F	K	E	Y	L	N	K	D	R
		*Melogale moschata*	S	F	K	E	Y	L	N	K	D	R
		*Arctonyx collaris*	S	F	K	E	Y	L	N	K	D	R
		*Enhydra lutris kenyoni*	S	F	K	E	Y	L	N	K	D	R
	*Otariidae*	*Eumetopias jubatus*	S	F	K	E	Y	L	N	K	D	R
		*Zalophus californianus*	S	F	K	E	Y	L	N	K	D	R
	*Phocidae*	*Neomonachus schauinslandi*	S	F	K	E	Y	L	N	K	D	R
*Rodentia*	*Cricetidae*	*Microtus oregoni*	S	F	K	E	Y	L	N	K	D	R
		*Microtus ochrogaster*	S	F	K	E	Y	L	N	K	D	R
		*Arvicola amphibius*	S	F	K	E	Y	L	N	K	D	R
	*Heteromyidae*	*Dipodomys ordii*	S	F	K	E	Y	L	N	K	D	R
	*Sciuridae*	*Sciurus vulgaris*	S	F	K	E	Y	L	N	K	D	R
	*Muridae*	*Grammomys surdaster*	S	F	K	E	Y	L	N	K	D	R
	*Dipodidae*	*Jaculus jaculus*	S	F	K	E	Y	L	N	K	D	R
*Artiodactyla*	*Lipotidae*	*Lipotes vexillifer*	S	F	K	E	Y	L	N	K	D	R
	*Phocoenidae*	*Phocoena sinus*	S	F	K	E	Y	L	N	K	D	R
	*Balaenopteridae*	*Balaenoptera musculus*	S	F	K	E	Y	L	N	K	D	R
		*Balaenoptera acutorostrata*	S	F	K	E	Y	L	N	K	D	R
	*Delphinidae*	*Tursiops truncatus*	S	F	K	E	Y	L	N	K	D	R
	*Physeteridae*	*Physeter catodon*	S	F	K	E	Y	L	N	K	D	R
	*Camelidae*	*Camelus bactrianus*	S	F	K	E	Y	L	N	K	D	R
	*Tayassuidae*	*Catagonus wagneri*	S	F	K	E	Y	L	N	K	D	R
	*Monodontidae*	*Monodon monoceros*	S	F	K	E	Y	L	N	K	D	R
		*Delphinapterus leucas*	S	F	K	E	Y	L	N	K	D	R
*Pholidota*	*Manidae*	*Manis pentadactyla*	S	F	K	E	Y	L	N	K	D	R
		*Manis javanica*	S	F	K	E	Y	L	N	K	D	R

**Table 6:** LCAS of potentially unsusceptible hosts.

Oder	Family	Species	19	28	31	35	41	45	53	68	355	357
*Primates*	*Cebidae*	*Saimiri boliviensis*	S	F	K	E	**H**	L	N	K	D	R
		*Sapajus apella*	S	F	K	E	**H**	L	N	K	D	R
		*Cebus imitator*	S	F	K	E	**H**	L	N	K	D	R
	*Tarsiidae*	*Carlito syrichta*	S	F	K	E	**H**	L	N	**I**	D	R
		*Condylura cristata*	S	F	**T**	E	Y	L	N	**M**	D	R
	*Aotidae*	*Aotus nancymaae*	S	F	K	E	**H**	L	N	K	D	R
	*Cebidae*	*Callithrix jacchus*	S	F	K	E	**H**	L	N	K	D	R
*Chiroptera*	*Pteropodidae*	*Rousettus leschenaultii*	S	F	K	E	Y	L	N	**T**	D	R
		*Rousettus aegyptiacus*	S	F	K	E	Y	L	N	**T**	D	R
	*Rhinolophidae*	*Rhinolophus ferrumequinum*	S	F	K	**K**	Y	L	N	K	D	R
		*Rhinolophus macrotis*	S	F	K	**K**	Y	L	N	K	D	R
*Rodentia*	*Muridae*	*Rattus norvegicus*	S	F	**N**	E	Y	L	N	K	D	R
	*Chinchillidae*	*Chinchilla lanigera*	**L**	F	K	E	Y	L	N	**L**	D	R
	*Bathyergidae*	*Heterocephalus glaber*	S	F	**N**	E	Y	L	N	**I**	D	R
	*Spalacidae*	*Nannospalax galili*	**L**	F	K	E	Y	L	N	**I**	D	R
	*Bathyergidae*	*Fukomys damarensis*	S	F	**T**	E	Y	L	N	K	D	R
*Artiodactyla*	*Camelidae*	*Lama glama*	S	F	**E**	E	Y	L	N	K	D	R
		*Camelus dromedarius*	S	F	**E**	E	Y	L	N	K	D	R
		*Camelus ferus*	S	F	**E**	E	Y	L	N	K	D	R
*Equidae*	*Equus*	*Equus przewalskii*	S	F	K	E	**H**	L	N	**R**	D	R
		*Equus quagga*	S	F	K	E	**H**	L	N	**R**	D	R
		*Equus asinus*	S	F	K	E	**H**	L	N	**R**	D	R
		*Equus caballus*	S	F	K	E	**H**	L	N	**R**	D	R
*Carnivora*	*Viverridae*	*Paguma larvata*	S	F	**T**	E	Y	**V**	N	K	D	R
	*Herpestidae*	*Suricata suricatta*	S	F	**Q**	E	Y	**V**	N	K	D	R
	*Otariidae*	*Callorhinus ursinus*	S	F	K	E	Y	**F**	N	K	D	R
*Tenrecs*	*Tenrecidae*	*Echinops telfairi*	S	F	**E**	E	Y	L	N	K	D	R
*Proboscidea*	*Elephantidae*	*Loxodonta africana*	S	F	**T**	E	Y	L	N	**R**	D	R
*Tubulidentata*	*Orycteropodidae*	*Orycteropus afer*	**A**	F	K	E	Y	L	N	**R**	D	R
*Diprotodontia*	*Vombatidae*	*Vombatus ursinus*	**F**	F	**T**	E	Y	L	N	**R**	D	R
*Perissodactyla*	*Rhinocerotidae*	*Ceratotherium simum*	S	F	K	E	Y	L	N	**R**	D	R

## 4. Discussion

We performed a comparative analysis of the ACE2 receptor-specific protein sequences of 109 species. The important 10 key amino acid sites that were commonly located in known SARS-CoV-2 susceptible species as reference standards for the analysis and used them to identify the potential risk host. The results reveal that 49 species were potentially susceptible hosts, and 31 species were non-susceptible hosts. Most of the potential susceptible hosts are distributed in the same order as the known susceptible hosts, indicating to some extent that closely related species are more susceptible. Particularly, two target species (Manis pentadactyla and Manis javanica), which appeared in the prediction results, have not been reported before. This indicates that while focusing on closely related species, it is necessary to pay attention to other target species and protect animals on a larger scale. The rising number of wild and domestic animals infected with SARS-CoV-2 challenges us to rethink outbreak control strategies in the post-epidemic era and prepare for future emerging infectious diseases.

However, not all closely related species are potentially susceptible. The key amino acids at position 41 of the ACE2 receptors in Capuchinidae, night monkeys, and marmosets differ from those in humans. A large number of studies have confirmed that 41-position amino acid mutations may break key hydrogen bonds, reducing the binding capacity of SARS-CoV-2 to ACE2 [17, 51]; Bats are generally considered to be the main natural hosts of the new coronavirus, but the 35 amino acids of *Rhinolophus macrotis* and *Rhinolophus ferrumequinum* of the *Rhinolophidae* family are different from humans [35]. The mutations in E35K can reduce the binding capacity of SARS-CoV-2. Jun Lan et. al. found that ACE2 of *Rhinolophus ferrumequinum* cannot mediate the entry of the new coronavirus [52]. It suggests that not all bats are susceptible to the new corona virus. Assessing the susceptibility of various bat species to the new coronavirus is the first step in the traceability process for bats, which can significantly reduce the challenges in tracing the new coronavirus. Paguma larvata, which showed inconsistency on LCAS, was not entirely consistent in the predictions, but recent studies have shown that it can be infected with the new coronavirus in vitro [18], which may be related to other factors inherent in the animal. Therefore, further research and analysis is needed on whether civet cats can be naturally infected and spread the new coronavirus.

In this study, a minimum number of key amino acid loci were selected based on the LCAS of known susceptible hosts, which greatly reduces the complexity of the work and allows for rapid and more accurate prediction of potentially susceptible hosts for the new coronavirus. Genetic variations in the host receptor ACE2 may also contribute to susceptibility or resistance against the viral infection, depending on how the variations in spike protein influence the cross‐species transmission of the virus. Studies have proved that after genetic mutations in S19, K31, E35, Y41, K68, and D355, the binding capacity of the virus to the receptor decreases [34, 35]. The predicted results are almost consistent with the results of other studies [26], indicating the accuracy of the results. The predicted results are almost consistent with the results of other studies [2], indicating the accuracy of the results. This method is simple and accurate, which can provide ideas to predicting the potential susceptible hosts in the early stages of disease outbreaks. It supports protective preventive measures for potential hosts in advance to control future outbreaks and reduce animal infections. The constant mutation of coronavirus increases its ability to bind to the ACE2 receptor as well as resist the immune response [53]. For example, N501Y can form a new interaction with the ACE2 receptor Y41, and it is widely present in mutants [54]. Especially the mutated Omicron strain S residue Y501 stacking interaction with the T-shaped π–π of Y41 in the ACE2 residue. The Q493R and Q498R mutations introduce two new salt bridges, such as E35 and E38, respectively replacing hydrogen bond formation and remodeling the electrostatic interactions with the ACE2 receptor of Wuhan-Hu-1 RBD. S477N leads to the formation of new hydrogen bonds between the asparagine side chain and the ACE2 S19 backbone amine and carbonyl groups [53, 55, 56]. These interactions illustrate that key amino acid sites on the ACE2 receptor are important for viral binding. we only considered key amino acid sites of virus-receptor interactions to predict susceptibility. However, the viral entry into host cells and replication were influenced by many other factors, such as cathepsin TMPRSS2 or CTSL1, and ADAM-17 [57]. Therefore, key amino acid sites alone are not sufficient.

## 5. Conclusions

In summary, we used a simple and accurate method to provide valuable insights into potential hosts at the early stages of disease outbreaks. We predicted 49 species as potentially susceptible hosts and 31 species as non-susceptible hosts. Notably, Manis pentadactyla and Manis javanica species were predicted, emphasizing the importance of considering a broader range of species in outbreak control. The research underscores the significance of genetic variations in the ACE2 receptor and how they influence susceptibility or resistance to viral infection. This information supports proactive preventive measures for potential hosts, aiding in outbreak control and reducing the risk of animal infections. However, it is crucial to acknowledge the study’s limitations and emphasize the ongoing need for research and validation to enhance our comprehension of cross-species transmission and preparedness for emerging infectious diseases. The prediction of SARS-CoV-2 infection risk species through key amino acid sites alone are not sufficient. Therefore, a comprehensive approach involving surveillance, laboratory validation, and clinical observation is essential to confirm the predicted potential susceptibility of animals to SARS-CoV-2 infection, crucial steps for controlling future outbreaks and contributing to a more nuanced understanding of cross-species transmission dynamics.

## Supporting information

S1 Graphical abstract (DOCX)
