# Supplementary figures and images for: Analysis the molecular similarity of least common amino acid sites in ACE2 receptor to predict the potential susceptible species for SARS-CoV-2
Source: PLoS One. 2024 May 2;19(5):e0293441. doi: 10.1371/journal.pone.0293441 (PMC11065212; doi:10.1371/journal.pone.0293441)

**Graphical abstract**

**
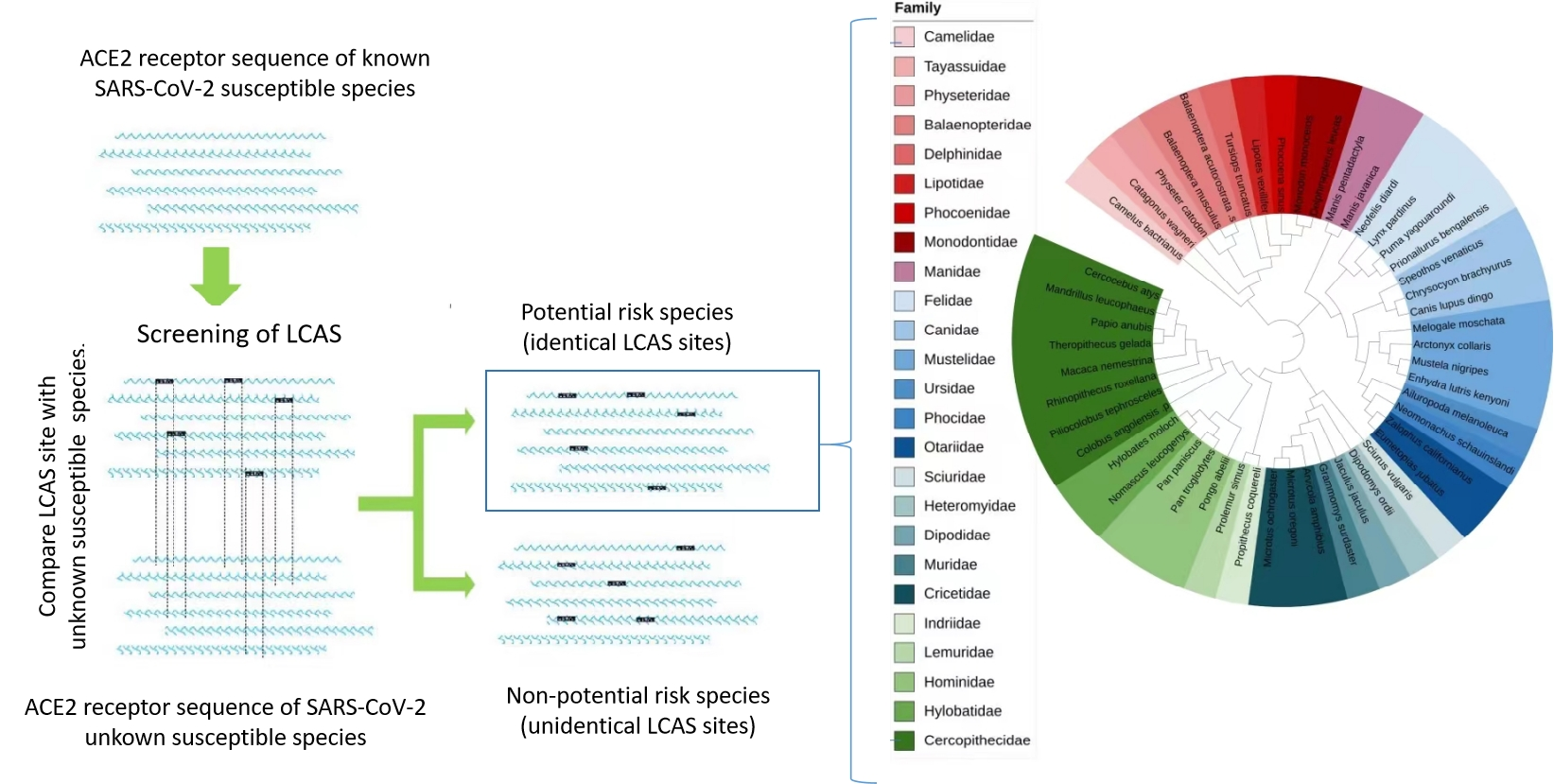
**

Supplement: S1 Graphical abstract — (DOCX) [file pone.0293441.s001.docx]
